# The use of GLP‐1 receptor agonists in hospitalised patients: An untapped potential

**DOI:** 10.1002/dmrr.3191

**Published:** 2019-06-28

**Authors:** Omar G. Mustafa, Martin B. Whyte

**Affiliations:** ^1^ Department of Diabetes King's College Hospital NHS Foundation Trust London UK; ^2^ Department of Clinical and Experimental Medicine University of Surrey Guildford UK

**Keywords:** acute illness, GLP‐1, inpatient, perioperative

## Abstract

In the outpatient setting, glucagon‐like peptide‐1 (GLP‐1) receptor agonists have proved to be highly efficacious drugs that provide glycaemic control with a low risk of hypoglycaemia. These characteristics make GLP‐1 receptor agonists attractive agents to treat dysglycaemia in perioperative or high‐dependency hospital settings, where glycaemic variability and hyperglycaemia are associated with poor prognosis. GLP‐1 also has a direct action on the myocardium and vasculature—which may be advantageous in the immediate aftermath of a vascular insult. This is a narrative review of the work in this area. The aim was to determine the populations of hospitalised patients being evaluated and the clinical and mechanistic end‐points tested, with the institution of GLP‐1 therapy in hospital. We searched the PubMed, Embase, and Google scholar databases, combining the term “glucagon‐like peptide 1” OR “GLP‐1” OR “incretin” OR “liraglutide” OR “exenatide” OR “lixisenatide” OR “dulaglutide” OR “albiglutide” AND “inpatient” OR “hospital” OR “perioperative” OR “postoperative” OR “surgery” OR “myocardial infarction” OR “stroke” OR “cerebrovascular disease” OR “transient ischaemic attack” OR “ICU” OR “critical care” OR “critical illness” OR “CCU” OR “coronary care unit.” Pilot studies were reported in the fields of acute stroke, cardiac resuscitation, coronary care, and perioperative care that showed advantages for GLP‐1 therapy, with normalisation of glucose, lower glucose variability, and lower risk of hypoglycaemia. Animal and human studies have reported improvements in myocardial performance when given acutely after vascular insult or surgery, but these have yet to be translated into randomised clinical trials.

## 1 INTRODUCTION

In the outpatient setting, GLP‐1 receptor agonists (GLP‐1 RA) are considered to provide effective glycaemic control, weight loss, and cardiovascular benefits with a low risk of hypoglycaemia.^1^, ^2^ The use of GLP‐1 RA have increased rapidly and are now recommended for use as second‐line therapy for type 2 diabetes (T2DM).^3^ As a result, individuals treated with GLP‐1 RA will be more frequently encountered at the inpatient setting. Existing clinical guidelines for inpatient diabetes control only briefly discuss their role, if at all.^4^, ^5^, ^6^ Furthermore, the therapeutic benefit for GLP‐1 RA is being tested in a range of acute clinical settings including perioperative, coronary care, and critical care.^7^, ^8^, ^9^, ^10^, ^11^, ^12^, ^13^, ^14^, ^15^ This narrative review will outline the inpatient clinical areas in which GLP‐1 RA are being tested, the panoply of actions of GLP‐1 that offer the potential for benefit in acute illness, and the outcome measures reported so far. This review will not encompass the effectiveness for GLP‐1 RA to reduce hospitalisation or mortality from macrovascular disease (such as heart failure) which has been covered extensively elsewhere^16^, ^17^; nor will it assess the usefulness of initiating GLP‐1 RA therapy in the months before surgery. GLP‐1 RA have been used with some success for short‐bowel syndrome^18^ but our analysis is restricted to the use of GLP‐1 for acute illness, perioperative, and critical care areas.

## 2 SEARCH METHODOLOGY

We conducted a comprehensive search in the English‐language literature to identify all relevant studies, regardless of publication status or year of publication. We searched the PubMed, Embase, and Google scholar databases, combining the term “glucagon‐like peptide 1” OR “GLP‐1” OR “incretin” OR “liraglutide” OR “exenatide” OR “lixisenatide” OR “dulaglutide” OR “albiglutide” AND “inpatient” OR “hospital” OR “perioperative” OR “postoperative” OR “surgery” OR “myocardial infarction” OR “stroke” OR “cerebrovascular disease” OR “transient ischaemic attack” OR “ICU” OR “critical care” OR “critical illness” OR “CCU” OR “coronary care unit.” Studies could include early or late phase human trials and those using animal models. We searched the National Institutes of Health database (http://clinicaltrials.gov/) and the EU clinical trials register (http://www.clinicaltrialsregister.eu) for ongoing and unpublished trials. We applied backward and forward snowballing to identify further papers. The bibliographies of all included studies and pertinent reviews were scanned for additional references. If required, the corresponding author of an included study was contacted for information regarding unpublished trials or complementary information on their own trial(s). The last search was performed in August 2018.

## 3 STROKE AND BRAIN INJURY

GLP‐1 RAs have certain properties that lend themselves to use in cerebrovascular disease: most obvious is the ability to normalise glucose with a low propensity to hypoglycaemia. Ischaemic stroke leads to a penumbra of neural tissue that is potentially salvageable. Hyperglycaemia is associated with worse clinical outcomes in stroke including haemorrhagic transformation, extent of neurological disability, and death.^19^, ^20^ Of those presenting with ischaemic stroke, 30% have known diabetes, 20% are newly recognised to have diabetes, and a further 30% have impaired fasting glucose or glucose tolerance.^21^, ^22^, ^23^ Therefore, just 20% have normal glucose homeostasis. Significant consequences arise from dysglycaemia: this may be due to the production of the excitatory neurotransmitter glutamate, or to reactive oxygen species which adversely affect the ischaemic penumbra. However, clinical trials in acute stroke have failed to show a clinical benefit from the amelioration of hyperglycaemia with insulin in the first 24 to 48 hours.^24^, ^25^, ^26^, ^27^ Neurological gains from the avoidance of hyperglycaemia are mitigated by hypoglycaemia from the use of insulin. Hypoglycaemia is associated with increased markers of cerebral cellular distress including elevated glutamate:lactate/pyruvate ratio: and glycerol—which may themselves contribute to disability and death.^28^, ^29^, ^30^, ^31^ There is therefore a J‐shaped association between plasma glucose and outcome after stroke.^32^ GLP‐1 RA offer the possibility of normalising glucose without hypoglycaemia.

In a pilot study of 11 patients with acute ischaemic stroke, subcutaneous exenatide (for a median of 6 days) restricted the incidence of hyperglycaemia (> 8.6 mmol/L) to less than 5%, with no hypoglycaemia. Of note, nausea and vomiting occurred in half the cohort,^7^ which may prove to be a limiting factor in future studies. A large phase 2 trial is underway: the Trial of Exenatide in Acute Ischaemic Stroke (TEXAIS) is a 3‐year, multicentre, open‐label randomised controlled trial (RCT) comparing exenatide with standard care. It aims to recruit 528 patients with a clinically meaningful primary end‐point of major neurological improvement at 7 days.^13^

Exendin‐4 is a 39 amino acid peptide originally isolated from the oral secretions of the Gila monster lizard. It shares 53% sequence homology with GLP‐1 and has been used in a number of preclinical studies.^33^ Murine studies of ischaemic brain damage using Exendin‐4^34^ and liraglutide^35^ show that they are capable of crossing the blood‐brain barrier to act directly on the brain to produce neuroprotective and anti‐inflammatory effects.^36^, ^37^ The neuroprotective properties that GLP‐1 RAs have shown in animal studies have also led to their evaluation in patients with brain injury after cardiac arrest. A dual‐centre study in Denmark has investigated the neuroprotective effects of exenatide administered within 4 hours of the return of spontaneous circulation to comatose patients resuscitated from out‐of‐hospital cardiac arrest.^14^ The efficacy endpoint was the area under curve (AUC) from 0 to 72 hours after admission of neuron‐specific enolase—a predictor of outcomes after cardiac arrest.^38^ Although exenatide was effective in normalising blood glucose (median blood glucose 8 hours after admission was 5.8 mmol/L vs 7.3 mmol/L, in placebo; *P* < .0001), exenatide did not reduce neuron‐specific enolase levels and did not significantly improve a composite end‐point of death and poor neurological function after 180 days.^14^ Of note, exenatide was not administered until after the return of spontaneous circulation which could limit the effectiveness. More recently, a 6‐hour infusion of either 17.4 μg of exenatide or placebo, within 4 hours from sustained return of spontaneous circulation, showed that exenatide lowered glucose and increased the clearance of lactate (a surrogate marker for adequate tissue perfusion^39^) by 21% more than placebo.^15^

## 4 MYOCARDIAL INFARCTION

Over 20 years after the “Diabetes Mellitus Insulin‐Glucose Infusion in Acute Myocardial Infarction” (DIGAMI) study, evidence for the necessity of tight glycaemic control in the immediate aftermath of AMI remains debatable.^40^ The DIGAMI study was primarily a trial of glucose‐insulin‐potassium (GIK) rather than glycaemic control and was predicated by the ideas that GIK may ameliorate platelet aggregation, reduce transmembrane excitability and therefore arrhythmias, and facilitate a switch in myocardial metabolism away from oxygen‐dependent fatty acid metabolism to carbohydrate metabolism. After the study, it could not be determined whether mortality benefit at 1 year was due to the immediate intravenous infusion of GIK, subsequent subcutaneous insulin, or both. It was hoped that the follow‐up DIGAMI2 would answer this but unfortunately was underpowered, in part due to protocol violations between groups and advances in therapeutic use of aspirin and statins.^41^ This results in a quandary as to glycaemic management after AMI. However, GLP‐1 RA have properties, in addition to glucose lowering, that make them attractive for use in the immediate aftermath of AMI. The GLP‐1 receptor is expressed in the heart,^42^ and exendin‐4 directly activates cardiomyocyte signalling pathways^43^ which raises the possibility they may act directly on the cardiac muscle to improve ventricular ejection fraction and cardiac index.^16^ GLP‐1 RA may also have an effect on increasing myocardial reliance on glucose, rather than fatty acid metabolism (thereby being more oxygen efficient), akin to that proposed for GIK, although this theory is contentious.^44^, ^45^, ^46^, ^47^ Altered myocardial metabolism has been reported with albiglutide. In a murine model, albiglutide reduced the myocardial infarct size in association with increased cardiac uptake and utilisation of glucose. Gene expression analysis indicated an upregulation of key glucose metabolism genes in the preserved myocardium post treatment.^48^

Intriguingly, GLP‐1 RA has a direct effect on myocytes independent to its action via the GLP‐1 receptor. Studies of isolated cardiomyocytes, from GLP‐1 receptor knockout mice, have shown a robust response to lixisenatide on the contractility response.^49^ Within the coronary vessels themselves, human recombinant GLP‐1 (7‐36) amide—the cleavage product of GLP‐1 (1‐36)—exerts a beneficial effect on endothelial function.^50^, ^51^ In coronary endothelial cells taken from subjects with T2D, Exendin‐4 could augment endothelial nitric oxide synthase (eNOS) phosphorylation and nitric oxide (NO) production^52^—pathways that are known to lead to vascular relaxation.^53^ Glucagon‐like peptide 1 [7‐36 amide]^54^ and liraglutide^55^ can exert a protective effect against endothelial dysfunction induced by hyperglycaemia and/or inflammation through a reduction of tumour necrosis factor‐α (TNF‐α)‐induced nuclear factor‐κB activation. This can decrease inflammatory gene expression, including vascular cell adhesion molecule‐1 and monocyte chemoattractant protein‐1.^56^ These properties have made GLP‐1 RAs an attractive treatment option for patients with acute coronary ischaemia, as was suggested from a number of animal models.^57^, ^58^, ^59^, ^60^ In 10 human subjects with left ventricular (LV) dysfunction, a 72‐hour infusion of GLP‐1 (7‐36) amide (1.5 pmol/kg/min), commenced after primary coronary angioplasty for acute myocardial infarction, resulted in improved global and regional LV wall motion scores and reduced hospital stay (6.1 ± 1.3 days) vs 9.8 ± 1.5 days in controls.^61^ There followed three publications by a group in Copenhagen, from a larger RCT (n = 172) composed of patients with ST elevation myocardial infarction (STEMI) treated with exenatide,^9^, ^10^, ^11^ both with and without diabetes. Improvements in infarct size were seen, but no mortality benefit, when administered intravenously during primary percutaneous coronary intervention (PCI).^9^, ^10^ Since then, most,^11^, ^62^, ^63^, ^64^ but not all,^65^ trials have reported an effect of reducing coronary infarct size. Differences in glycaemic control achieved by the intervention may go towards explaining the difference. The protocol used by the Copenhagen group^9^, ^10^, ^11^ comprised exenatide or placebo given intravenously 15 minutes prior to intervention and continued 6 hours post‐PCI—at which time blood glucose was 8.0 mmol/L in controls and 6.3 mmol/L with exenatide. Conversely in a study by Roos et al,^65^, ^66^ exenatide was administered for 72 hours after primary angioplasty in 91 individuals, with blood glucose measured every 3 hours on day one, reduced to four‐times per day thereafter; there were no differences in the number of hypoglycaemic episodes (considered <4 mmol/L [10 events in exenatide vs 9 in placebo; 0.530]) or hyperglycaemic episodes (considered >10 mmol/L [3 events in exenatide vs 10 in placebo; 0.064]).

The use of GLP‐1 RA in non‐ST elevation myocardial infarction (nSTEMI) was examined in a study of 90 patients randomised to liraglutide (0.6 mg for 2 days, 1.2 mg for 2 days, followed by 1.8 mg for 3 days) or placebo for 7 days; liraglutide improved LV ejection fraction by 4.7% more than placebo after 3 months.^67^ However, this seems a modest improvement and may not translate into measurable clinical outcomes. A significant improvement has been defined as an increase in the ejection fraction of ≥8% (which is two times the interobserver variability with echocardiography).^68^

Is it feasible to achieve rapid normalisation of glucose after myocardial infarction with GLP‐1? This was addressed in a study of 40 patients admitted to a CCU with hyperglycaemia ranging from 7.8 to 22.2 mmol/L.^69^ Patients received intravenous exenatide as a bolus followed by a fixed dose infusion for up to 48 hours. Exenatide effectiveness was benchmarked to historical controls treated with insulin infusions. There was no difference in performance in the attainment of a target glucose range of 5.6 to 7.8 mmol/L although exenatide was discontinued in three patients after failure to achieve glycaemic control. Control was achieved without any episodes of severe hypoglycaemia (<2.8 mmol/L) although nausea (occurring in 16 patients) was problematic. By far, the largest trial to date has been the Evaluation of LIXisenatide in Acute coronary syndrome (ELIXA) study.^70^ This randomised 6068 patients within 180 days of a cardiovascular event that required hospitalisation. Once‐daily subcutaneous injection of 20 mcg lixisenatide, as an add‐on therapy to background antidiabetic medications, for 25 months was noninferior to placebo for the composite occurrence of cardiovascular death, nonfatal MI, nonfatal stroke, hospitalisation for unstable angina or heart failure, and revascularization. This study provided data for cardiovascular safety of lixisenatide but no advantage. The 6‐month window from cardiovascular event to recruitment does, however, make it distinct from the trials described.

## 5 CARDIAC SURGERY

Perioperative glucose control reduces the incidence of sepsis and mediastinitis after cardiothoracic surgery.^71^, ^72^ Van den Berghe's 2001 paper,^73^ reporting improved postoperative outcomes with tight glycaemic control after (predominantly cardiovascular) surgery, continues to be hotly debated as the findings have not been replicated in multicentre trials.^74^, ^75^, ^76^, ^77^ There is an increased likelihood of hypoglycaemia from the intensification of glucose targets with insulin therapy, which leads to worse outcomes.^74^, ^76^, ^78^ GLP‐1 therapy offers the prospect of glycaemic control with less likelihood of hypoglycaemia after coronary surgery which may tip the balance in favour of tighter glycaemia targets when using this drug. The perioperative administration of exenatide (1.2‐1.5 pmol/kg/min) has proved to be effective at improving glucose levels in patients with diabetes and stress hyperglycaemia undergoing cardiac surgery and can reduce perioperative insulin requirements,^79^, ^80^, ^81^ although reduction in hypoglycaemia was not always observed.^80^ One trial in subjects with T2DM used a much higher infusion rate (3.6 pmol/kg/min) for 12 hours after transfer from the operating room to the intensive care unit (ICU)^82^ combined with insulin as a rescue medication if glucose concentrations were > 7.8 mmol/L, for over 3 hours. The AUC for plasma glucose was no different between controls (receiving insulin alone) and exenatide. Compared with patients receiving intravenous insulin, a lesser insulin requirement, with fewer dose adjustments, was necessary in the GLP‐1 group over the first 6 hours.

In a small study of 20 patients with coronary heart disease and preserved LV function who underwent CABG, use of GLP‐1 [7‐36 amide] as a continuous infusion beginning 12 hours before CABG and continuing for 48 hours resulted in less requirement for inotropic support—even in patients without diabetes.^83^ However, no improvement in echocardiographic features of LV dysfunction was seen in this study, or in a similarly executed study of perioperative intravenous exenatide with cardiothoracic surgery.^81^, ^83^ These postoperative trial outcomes corroborate the findings post MI suggesting little role for exogenous GLP‐1 to improve cardiac performance acutely.

The “GLP‐1 for bridging of hyperglycaemia during cardiac surgery” (GLOBE) study is a large randomised parallel placebo‐controlled trial, currently underway, with the intention of recruiting 274 patients undergoing cardiac surgery, with or without diabetes mellitus.^84^ Patients will receive 0.6‐mg liraglutide or placebo the evening before and 1.2‐mg liraglutide or placebo just prior to surgery. The primary endpoint is intraoperative insulin requirement: a relevant outcome as greater insulin infusion rates are associated with adverse clinical sequelae.^73^, ^85^ Taken together, these studies suggest that perioperative GLP‐1 helps to achieve glycaemic control, with a lessened requirement for insulin. Further evidence is required whether this translates into fewer hypoglycaemic episodes. The immediate haemodynamic benefits are unproven.

## 6 GENERAL SURGERY

Less work has been undertaken in noncardiac surgery. In a proof‐of‐concept study, eight patients with T2DM who had undergone major surgical procedures were studied with a cross‐over design, between the second and the eighth postoperative day.^86^ Patients received intravenous GLP‐1 (7‐36) amide (1.2 pmol/kg/min) or placebo over 8 hours, each administered in randomised order in the fasting state. From a fasting glucose of 10 mmol/L, infusion of GLP‐1, lowered plasma glucose concentration to target (<7 mmol/L) within 150 minutes, whereas glucose remained above target throughout the 8‐hour placebo infusion (*P* < .001). Rapid attainment of normoglycaemia in the postoperative period makes GLP‐1 an attractive option. Data are needed as to the efficacy of simple subcutaneous regimes.

For patients undergoing hip surgery, perioperative hyperglycaemia is associated with coagulation activation and an increased risk of venous thromboembolism.^87^ The effect of liraglutide on markers of coagulation has been tested in obese adults without diabetes, over the first 3 days after hip surgery. Despite improvement in median glucose (5.5 mmol/L vs 5.8 mmol/L with placebo; *P* = .04), there was a negligible change in the markers of coagulation activation.^88^ Overall, there are few data to support the specific indication of GLP‐1 RA use with general surgery.

## 7 INTENSIVE CARE UNIT

The challenge of maintaining blood glucose between the hazards of hyperglycaemia and hypoglycaemia is equally true in ICU as it is with perioperative patients. Tight glycaemic control (4.4‐6.1 mmol/L) significantly increases the risk for hypoglycaemia, which can lead to poor outcomes.^74^, ^76^ The administration of GLP‐1 (7‐36) amide has been tested for its glucopaenic action at the time of ICU admission. In a parallel‐design RCT, 72‐hour infusion of GLP‐1 did not reduce intravenous insulin requirement nor the rate of hypoglycaemia compared with saline control, although there was less plasma glucose variability with GLP‐1. Low glucose variability is protective in ICU patients, even when mean glucose levels are elevated,^89^ making this a potential attribute of GLP‐1 therapy in the ICU environment.

Of note, this study did not control for the routes or quantity of nutritional support.^8^ An open‐label RCT in India evaluated the effect of commencing fixed‐dose liraglutide (1.2 mg) from the time of ICU admission: in a study population of 120 individuals, 46 were considered to have preexisting T2DM and another 53 stress‐hyperglycaemia. Intravenous insulin infusion was also used as required. Liraglutide reduced hypoglycaemia frequency and the variation of capillary glucose but with no difference in mean capillary glucose.^90^ An intriguing line of inquiry is whether GLP‐1 can also ameliorate ICU catabolism. Critically ill patients in ICU suffer significant muscle loss. This worsens ICU mortality but also leads to debilitating weakness in those surviving to ICU discharge.^91^ Hyperglucagonaemia is thought to be a key factor to provoke catabolism and hyperglycaemia in critical illness.^92^, ^93^ Given that a core attribute of GLP‐1 is to suppress glucagon release from pancreatic alpha‐cells,^94^ GLP‐1 has theoretical anticatabolic action as well as being effective in ameliorating stress hyperglycaemia.^86^, ^95^

A further challenge in prolonged critical illness is to maintain normoglycaemia in the setting of enteral and parenteral feeding protocols. A number of mechanistic studies from a group in Adelaide, Australia have shown that an infusion of GLP‐1‐(7–36) amide at 1.2 pmol/kg/min attenuates, but not abolishes, the glycaemic response to enteral nutrition in critically ill patients with stress hyperglycaemia^95^, ^96^ and with T2DM.^97^ Slowing of gastric emptying appears to contribute to the glucose‐lowering effect of exogenous GLP‐1 in critically ill patients following a 100‐mL intragastric “meal.”^96^ However, delayed gastric emptying is common in the critically ill and may occur in up to half of all ventilated patients.^98^ This may limit the magnitude of glycaemic control that could be achieved with GLP‐1 RA during enteral feeding.^96^ Even so, there may be the potential for benefit even in patients fed parenterally. In a cross‐over study of nine critically ill patients fed with parenteral nutrition consisting of glucose (3.2 ± 1.4 mg/kg/min), amino acids (n = 8; 0.9 ± 0.2 mg/kg/min), with or without lipid emulsions, 4‐hour infusion of GLP‐1‐(7‐36) amide lowered glucose from 11.7 ± 1.3 mmol/L (with placebo) to 8.8 ± 1.4 mmol/L (*P* < .001).^99^ Nausea is often encountered with GLP‐1 RA but may be less of a concern in sedated patients receiving small intestinal feeding. Further issues to contend with in critical illness are kidney failure, pancreatitis, and the use of vasoactive drugs (including catecholamines) leading to a counter‐regulatory hormonal response.

## 8 CONCLUSION

GLP‐1 RA have a number of properties in addition to glucose lowering, which could be advantageous when started in an acute, hospitalised setting. However, there are few trial data in human subjects to support their adoption in routine clinical practice in this environment. The increasing use of GLP‐1 RA in outpatients will mean that patients using these medications will become increasingly seen in acute settings. Trials are needed to establish their place in acute illness.

## CONFLICT OF INTEREST STATEMENT

M.B.W. has received research funding from Eli Lilly and Sanofi, speaker fees from Astra Zeneca and Merck Sharp & Dohme (MSD), and consulting fee from Boehringer‐Ingelheim. O.M. has no conflict of interest.

## FUNDING

No funding was received for this article.
